# The state-of-art of design and research for Pelton turbine casing, weight estimation, counterpressure operation and scientific challenges

**DOI:** 10.1016/j.heliyon.2021.e08527

**Published:** 2021-12-03

**Authors:** Emanuele Quaranta, Chirag Trivedi

**Affiliations:** a European Commission Joint Research Centre, Ispra, Italy; b Waterpower Laboratory, Faculty of Engineering, NTNU—Norwegian University of Science and Technology, Trondheim, 7491 Norway

**Keywords:** Counterpressure, Casing, Fluid structure interaction, Hydropower, Pelton, Turbine

## Abstract

The Pelton turbine is the most widespread and efficient impulse hydropower turbine. The Pelton casing is a static, but key component: the internal hydrodynamic phenomena affect the performance of the hydropower plant, the vibration of the equipment and water quality (dissolved oxygen downstream). However, the literature information is very fragmented and not well organized, so that the design is generally based on empirical rules and on proper know-how of hydropower companies. In this paper, the state-of-the-art of the Pelton casing is reviewed and organized under three macro areas: hydraulics, mechanics (vibrations and weight) and aeration. The preliminary design procedure is described and discussed in light of recent scientific results, and the open questions and research challenges are highlighted. Innovative case studies are described (including counterpressure operation) and a dataset of installed casings (not available in literature) is elaborated to derive an empirical equation to estimate the casing weight. The efficiency can be improved by 3% by an optimal fluid dynamic design and a better understanding of the internal hydrodynamics. Proper inserts can improve the hydraulic efficiency by 2%, reduce the weight (by about 12%) and better bear the vibrations. Several scientific questions are still open, and a better understanding of the fluid structure interaction is needed to improve efficiency, operation and water quality.

## 1 Introduction

The global installed capacity of hydropower reached 1330 GW in 2021, and it will need to grow by around 60% by 2050 to deal with the increasing energy demand [1]. Although hydropower technology is quite mature, several improvements are under development to improve its flexibility, reliability and sustainability (e.g. reduction of environmental impacts), with also focus on the hydraulic turbine [2, 3].

**Figure 1:**
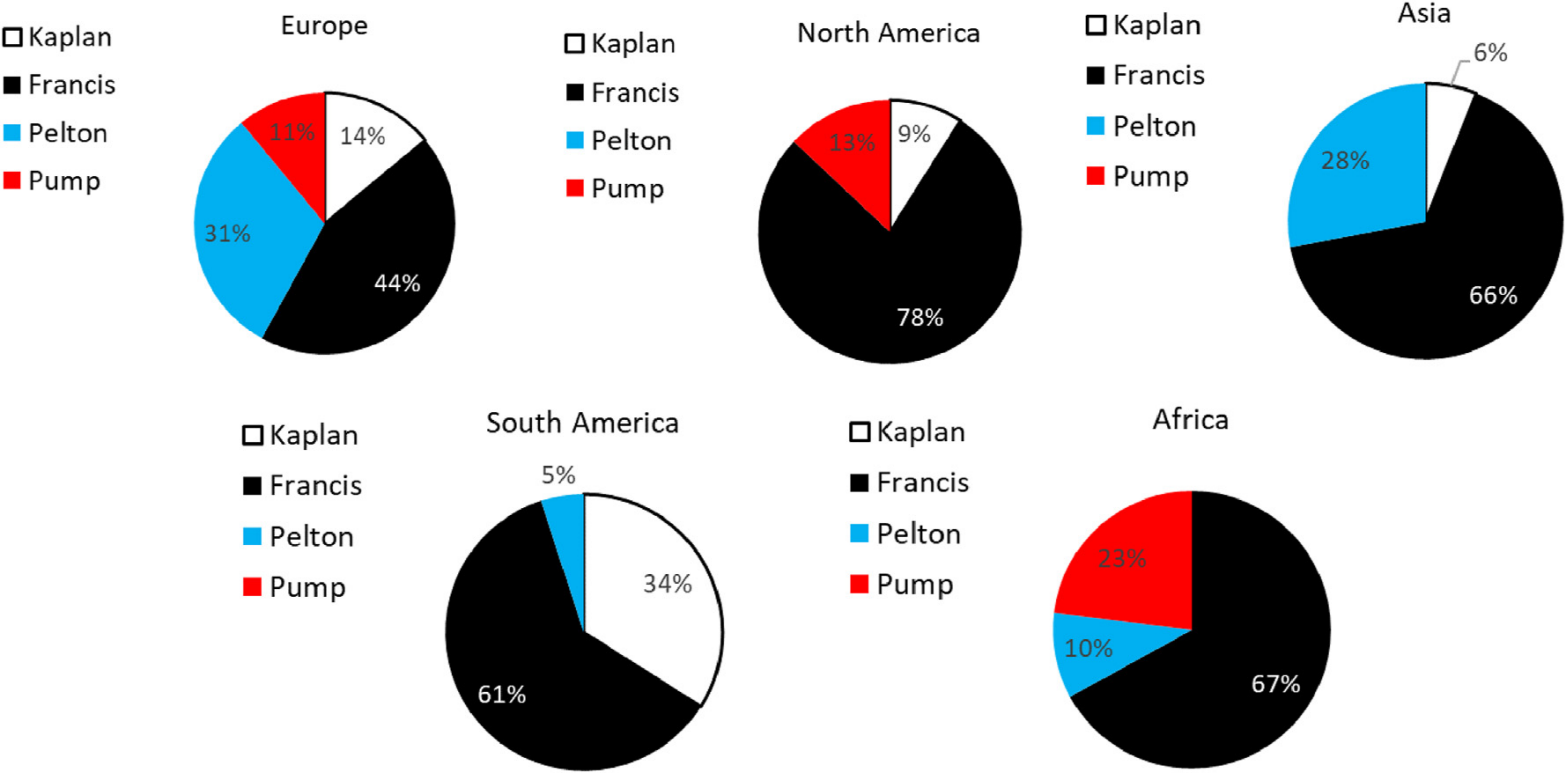
Turbines larger than 50 MW across the world. Data from [9].

Among the hydraulic turbines, the Pelton turbine, invented by Lester A. Pelton in 1880, is the most advanced impulse turbine. It generates power by utilizing the momentum of a water jet that impinges (tangentially) on the buckets mounted on the periphery of the runner. Although the highest efficiency can reach 92% [4], the Pelton turbine continue to be improved [5, 6, 7]. 16% of the current installed turbines in the European Union are Pelton turbines [8], while in Europe 31% of turbines in hydropower plants larger than 50 MW are Pelton type (Figure 1, data of 2009).

**Figure 2:**
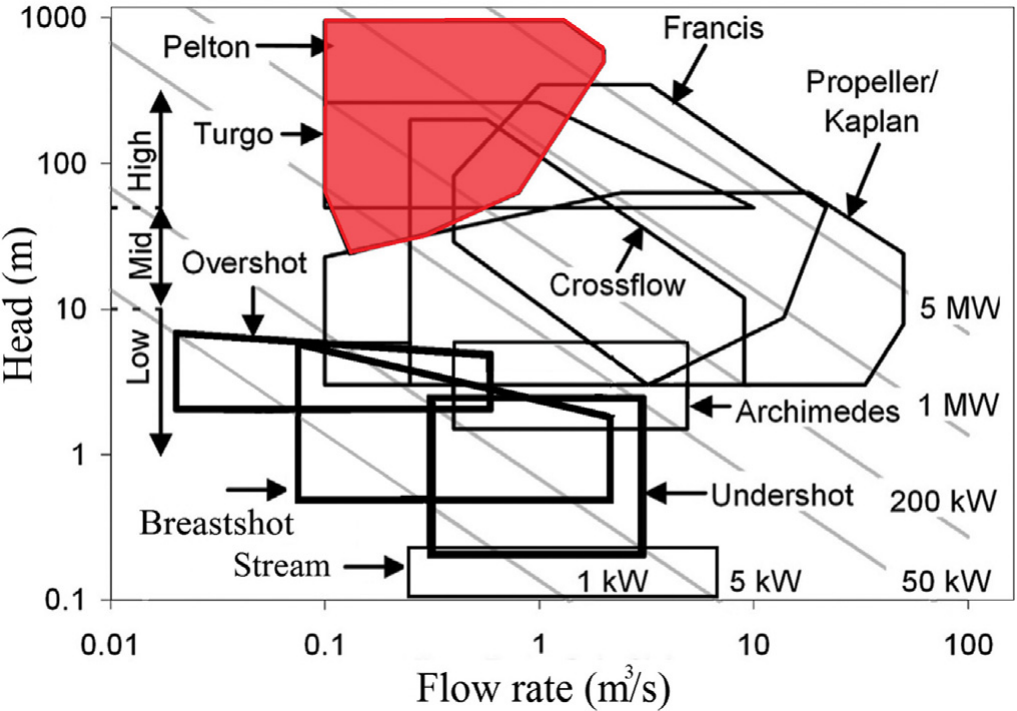
Operating range of common turbines, adapted from Quaranta and Revelli (2018) [11].

For a certain power *P*, Pelton turbines are employed at higher head sites and lower flow rates with respect to reaction turbines (i.e., Kaplan and Francis turbines), thus Pelton turbines are typically used in mountain regions. Their operating range is shown in Figure 2 and compared with that of the other turbines considering *P* > 50 kW. When *P* < 50 kW, micro Pelton turbines (micro hydropower is generally used when the installed power is below 100 kW) can be implemented in particular conditions of high heads and low flows, e.g. in aqueducts and with residual/ecological flows from dams [2], with a head ranging from 10 m to 300 m and a minimum flow of 0.5 l/s to guarantee a minimum jet diameter > 4 mm (smaller jet diameters are not recommended due to the difficulty to concentrate the jet and to minimize friction losses [10]).

The water flow rate is regulated by a nozzle (or injector) that at part load can maintain the efficiency above 90%, ideally until the flow rate is reduced at 20% of the design flow rate [12]. The turbine is enclosed inside a casing. A deflector is installed to stop the water jet in case of damages or emergency [13, 14].

Pelton turbines can be with horizontal axis or vertical axis. In the horizontal axis configuration, no more than two injectors can be installed, while vertical axis turbines can be provided with up to six injectors, which are symmetrically mounted around the wheel. When one injector is used, and in horizontal axis turbines, the one-sided bearing load can be significant. For turbines with more injectors, collisions between two jets must be avoided. The axis configuration also affects friction and windage losses [15].

Guidelines to design Pelton turbines are standardized and can be found in textbooks (e.g. [12, 15, 16, 17, 18]), while recent scientific studies provide more insights for further optimization strategies, e.g. the 3D shape of the bucket [19, 20, 21], and jet quality [13]. The design of Pelton units continues to improve, especially with the help of computational fluid dynamic (CFD) simulations [21, 22, 23, 24].

One of the essential components of a Pelton unit is the runner casing. Although it is a static component, its design is of extreme importance for the whole performance of the plant. The casing must (1) guarantee an optimal fluid dynamic behavior to maintain high efficiency, (2) support the runner and the vibrations related to the fluid-structure interaction, (3) ensure minimum ventilation losses, and (4) maintain a good aeration [25, 26]. The need to satisfy all these tasks makes very difficult to find an optimal standardized design, and available data are very limited in the literature. Standard procedures to optimize the casing from the fluid dynamic perspective have been neither critically designed nor optimized. The literature is in fact quite fragmented and several research and engineering questions are open and yet to be addressed [27].

Therefore, this paper presents a critical review of the Pelton turbines, specifically emphasizing on the casing component. The first section reviews the available guidelines for casing design. The second section discusses casing hydrodynamics, while the third section analyses aeration related topics. A section on the structural analyses (weight and vibration) is presented, with a list of real installations that were elaborated to determine an empirical equation for the weight estimation. In each section, the literature review aims to generalize and compare results, proposing guidelines for potential studies. Open challenges are also presented and comparisons with similar technologies are discussed to stimulate future scientific work.

## 2 Casing design: engineering characteristics and available guidelines

**Figure 3:**
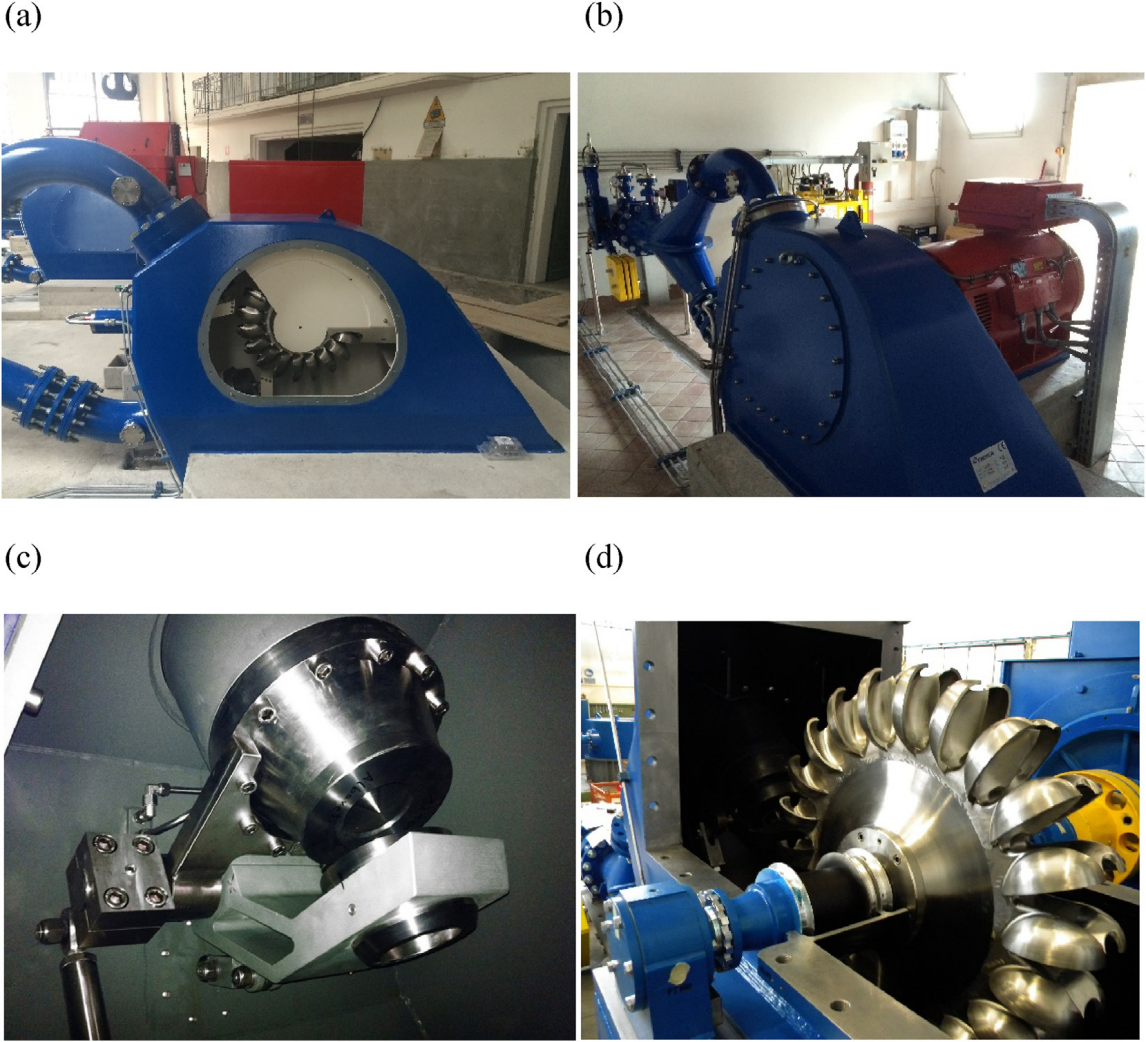
(a) 4 MW Pelton turbine, Miralago Valcanale; (b) 100 kW Terzago plant; (c) Doble needle of 1.6 MW plant (a,b,c: photo courtesy of Artingegneria); (d) and Pelton turbine of Zeco Hydropower.

The casing houses the runner and often supports the generator (Figure 3). An adequate hydraulic design is required to guide the water flow and to prevent the water, leaving the buckets, be deflected back against the runner, reducing turbine efficiency (more details are presented in the section of *Hydrodynamics and Fluid Dynamic optimization*). An adequate mechanical design is also needed to support the weight of the generator, the forces developed in the pipes, the forces generated by the jets, the load of the actuation mechanisms and vibrations (see section *Structural behavior***)**. The aeration is also of extreme importance to reduce ventilation losses and to ensure an optimal oxygen content (see section *Aeration*).

The casing cross section can be polygonal or circular (easier to be manufactured and with a smooth profile). Manufacturing aspects play important roles, and must consider the size, manufacturing complexity, weldability, possibility of corrosion, easy to dismantle for inspection and replacement of runner/bucket.

The casing is casted (old casings) or welded (new casings), and stiffened by ribs. Shrouds and baffles are added to properly guide the water flow into the tailrace to minimize the interference with the water jets. If serial production of Pelton turbines is not planned, the casing can be made as a welding of sheet metal, avoiding the cost of mold/cast. However, for the fabrication of the buckets, casting, and sometimes forging, facilities are required, so that the casting of the casing is often the chosen solution [10].

The casing is generally supported on a frame anchored by bolts to the concrete structure or embedded in concrete [15]. To dampen the vibrations, concrete masses are mounted on the surrounding of the branch pipe/manifold, covering the steel casing, to increase the weight of the substructure. The branch pipe/manifold is either mounted on the casing or embedded in the concrete [18]. Low carbon steel has been generally used for casing manufacture, and the structural design procedure should aim at maximizing the rigidity instead of minimizing the stresses [28].

Generators can be attached to the casing or separately supported. For vertical axis turbines, the cheapest solution is when the generator is fixed directly on the casing and the rotor is mounted on the generator shaft. Two bearings, one seal and a coupling can be eliminated with this design, but there is not possibility to install a flywheel. Instead, horizontal axis turbines are generally equipped with 1–2 jets and are well suited for the installation of one or two flywheels, in order to reduce speed variations [10].

Textbooks on hydraulic turbines generally suggest empirical equations to design the external casing dimensions, e.g. Eqs. (1), (2), (3), (4), (5), and (6). The casing diameter *D*_*c*_ should be at least 2.5 times the runner external diameter *D*_*r*_ to avoid impact and deflection of water. The height of the portion of casing above the jet *h*_*n*_ (in case of vertical axis) should be designed to avoid jet interference with the solid walls. The casing width varies with the distance from the axis. For horizontal axis turbines, [29] summarizes the width suggested from historic literature. Below the jet, the width of the casing *B*_*c*_ should be 11–18 jet diameters. Above the jet, there is not agreement, and the width ranges between 3-5 jet diameter [30] and 11–13 jet diameter. The bottom portion of the casing should be large enough to insure free discharge from the buckets. Near the contact point between the jet and the bucket, the width of the casing *B*_*c*_ should be 12–18 jet diameters [12, 16, 18].

(1) *D*_*c*_ = 2.5 *D*_*r*_

(2) *h*_*n*_ = 0.45*D*_*r*_

(3) *h* = (0.5÷1.0) + 0.5*D*_*r*_

where *h* is the distance between the runner centerline (vertical axis type) and the free surface of the tailrace. In vertical axis turbines, when the side walls taper upwards by about 10°, the water flows down helically [10].

de Siervo and Lugaresi (1978) [14] proposed similar equations:

(4) *D*_*c*_ = 0.78 + 2.06 *D*_*r*_

(5) *h*_*n*_ = 0.196 + 0.376*D*_*r*_

(6) *h =* 0.47 + 0.2 *D*_*c*_*=* 0.63 + 0.41*D*_*r*_

The units of the above equations should be selected coherently in matrix.

## 3 Hydrodynamics and fluid dynamic optimization

### 3.1 Complexity and research challenges

**Figure 4:**
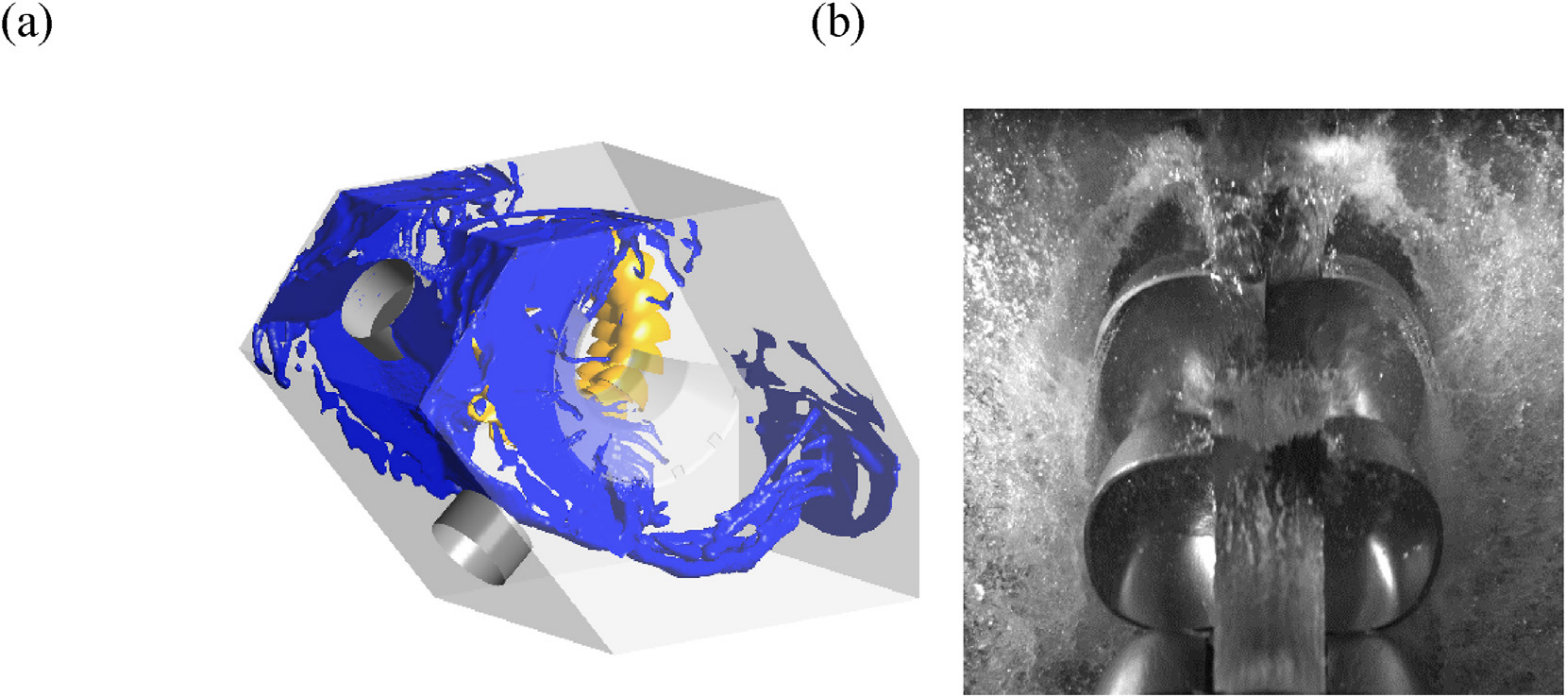
Flow field in a Pelton casing (a) (photo courtesy of Sean Petley, CFD simulation) and focus on the jet (photo courtesy of Bjorn Winther Solemslie).

The flow in a Pelton casing is very complex, because it is multiphase (see section *Aeration*), turbulent and unsteady. Different length scales are involved, from the main jet, that is comparable with the turbine bucket width, to small water droplets, and with a velocity that can be one order of magnitude lower than the main jet velocity (Figure 4 [31]). The ventilated droplets, water centrifuged from the buckets and splashing water diverted from deflectors, make the process complex by interfering with the jet. The highly pressurized air bubbles may remain enclosed in the oncoming water, and explode during their expansion after the nozzle exit. This is a common phenomena during the starting process of Pelton turbines, that can be easily heard outside of the casing during start-ups [32, 33]. The high Reynolds and Weber numbers are responsible for the further formation of fog in the casing, due to the small droplets [34].

The correct understanding of the flow allows to better calculate the stresses in the inserts and to reduce the jets interferences with the buckets. Insofar, there has been limited focus on investigating the flow field in the casing or related with the casing design [35]. Experimental tests are generally not effective because the air in the casing becomes rapidly saturated, hiding flows and jets, especially those far from the casing walls [36]. Eulerian and Smoothed-particle hydrodynamics (SPH) computational methods have been used to simulate casing flow, but with some limitations. The main limitation is the flow complexity as stated above and the requirement of very high number of mesh points in Computational Fluid Dynamic (CFD) simulations. Requirement of mesh and longer simulation time are the key factors for including/excluding the casing domain for CFD analysis. Droplets are difficult to be captured. The influence of the unpredictable flow patterns on the overall efficiency of the machine is expected to be in the range of the other numerical and physical errors that are introduced into the simulation [37]. These limitations pose the light on the need of better improving the research in this context and of better understanding the available literature results.

### 3.2 Literature review

The flow in a Pelton casing is generally driven by inertia; the water is guided to the tailrace by the casing walls and inserts. Inserts are installed to regularize the flow and to prevent undesired impacts of the water against solid surfaces, which can decrease the efficiency of the turbine. Matthias et al. (1997) [38] made a systematic classification of the flow distribution by observing the flow in a rectangular casing:

1. at low rotational speeds (low unit speed – optimal unit speed) the pattern is independent of the flow;
2. in the optimal range of operating rotational speeds (optimal unit speed – high unit speed) the pattern is independent of the flow;
3. at rotational speeds higher than the optimal, the pattern is dependent on the flow;
4. above the runaway speed of the turbine, no covering of the turbine with splash water can be observed.

Petley and Aggidis [27, 29, 39] performed CFD simulations to investigate the flow field inside the casing. The tests were carried out using the twin jet Z120 Pelton manufactured by Gilbert Gilkes & Gordon Ltd, which was coupled to a 75kW DC generator with continuous speed regulation. Realizable k-ϵ turbulence model and the Volume of Fluid (VOF) multiphase model were adopted to model turbulence and free surface. Power losses in the casing were quantified around 3.3% in the worst case (narrowest casing). The optimal casing width *B*_*c*_ was found to be 520 mm for a bucket width *B* = 120 mm, thus *B*_*c*_ = 4.33 *B*, although also *B*_*c*_ = 420 mm could be considered an optimal compromise. In [29] more details are provided, showing that the twin jet configuration generally exhibits an increased efficiency of 0.2–0.5 percentage points with respect to the single jet configuration.

**Figure 5:**
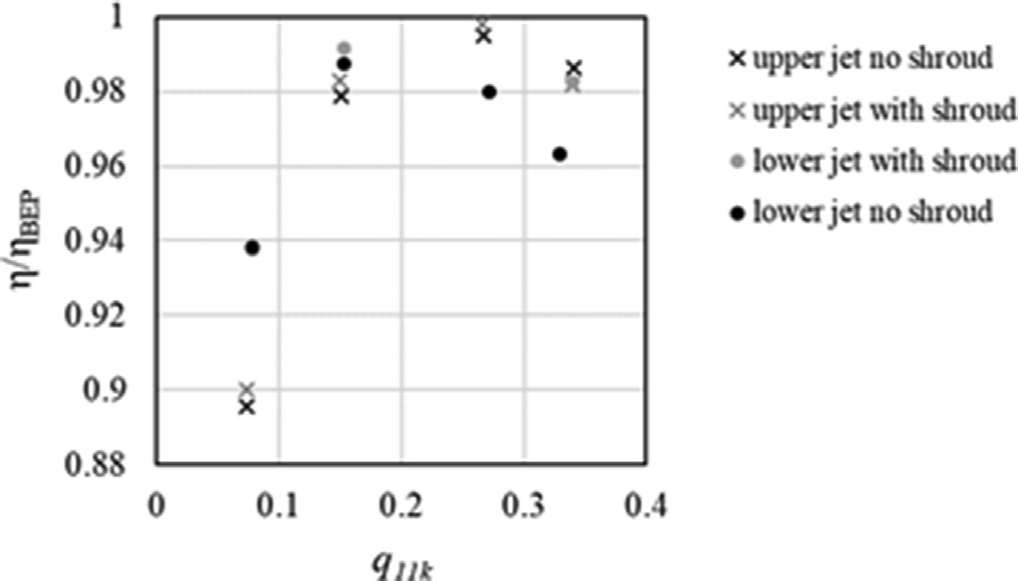
Efficiency versus unitary flow rate q_11k_ = *Q B*^−2^*H*^−0.5^ (*B* = bucket width), elaborating data of [27].

Without shrouds or baffles, the water circulates around the casing; in order to avoid that the circulating water interferes with the jet, in [27] a side shroud was placed over the injectors of a horizontal axis turbine, improving the performance (Figure 5). Nevertheless, the efficiency was more affected by the casing width (3.3% maximum improvement at the BEP by enlarging the casing) than by the presence of shrouds (2%) [27]. Furthermore, a curved baffle can be placed around the runner to avoid water entering the casing roof, ensuring that the water from the lower jet is deflected by the curved shape downwards and flows into the tailrace with minimal further interference. This allowed a performance improvement by 0.52%.

In [13] the optimization of the hydro power plant of Rothenbrunnen was presented. This plant is located in the Eastern Alps of Switzerland near the city of Thusis, where the compensation reservoir of Safien Platz supplies water. The gross head is approximately 670 m. Three double (two runners for each turbine) Pelton turbines with horizontal axis were installed in the mid of the 1950s by the Swiss company of Charmilles, with one nozzle per runner. The power output of the turbine before refurbishment was 42 MW, that was improved by 1.4% by replacing the injectors and modifying the casing after a careful study of the jet quality and splashing water development in the casing, reducing the number of droplets and jet dispersion.

Also Matthias et al. (1997) [38] conducted a fluid dynamic optimization on a horizontal axis turbine with 2 jets, improving the efficiency by 2.8% by optimizing the casing. It was found that at high flows the casing should be designed with a very small slit between the wall and the runner, or a great radial and axial expanse of the dome.

Two auxiliary wheels on either side of the central main runner may be used to further extract the residual kinetic energy of the water jet exiting the main buckets and to minimize water splashes on the casing walls [40].

## 4 Aeration

### 4.1 Complexity and research challenges

The optimal aeration inside the casing is essential to avoid depression in the casing, which may generate a rise of the tail water level and a deficit of oxygen downstream [26]. The natural aeration system should aim at reducing the need of forced air, minimizing costs, e.g. the cost of compressors (the volumetric air flow provided by compressors generally ranges between 6% and 60% of the water flow [41]). For example, the largest Pelton turbines in the world (in Bieudron, hydraulic head 1,869 m and design flow rate 25 m^3^/s), were designed to supply naturally 6 m^3^/s of air to the turbine casing [42]. However, little is known about the influence of overpressure on air/water mixtures produced by a plunging free jet, as occurring in Pelton turbines [43]. One additional challenge is the understanding of the detrainment of air that might occur during counterpressure operation (also called backpressure) operation [41].

### 4.2 Literature review

In [44] a new runner design was provided for the Tillari power plant. Several turbine casing modifications were tested and the optimal turbine casing shape was determined. The optimization of position and size of the sealing gap improved turbine efficiency up to 10 percentage points (Figure 5 of [44]). The sealing gap is important for natural air admission to the turbine casing and to prevent the water flow to the turbine bearing.

**Figure 6:**
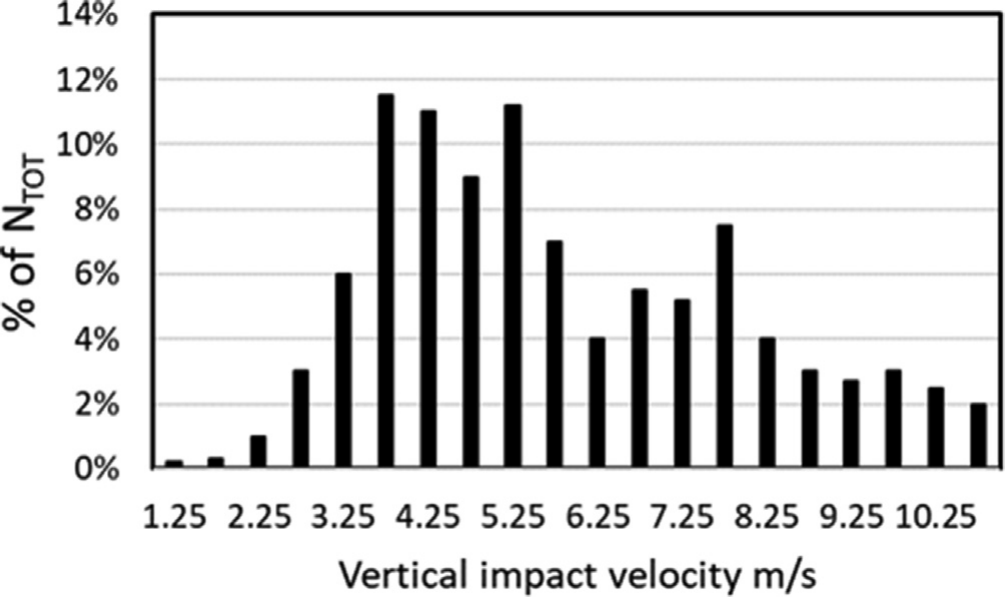
Distribution (N_TOT_ = total number of droplets) of water droplet velocity, adapted from Arch and Mayr (2012) [36].

Measurements were also performed in [36] on a Pelton turbine under *H* = 710 m head. It was found that near to the casing walls the flow velocity was lower than the value corresponding to the free fall velocity, probably due to the friction losses at the casing wall and the interaction of the several water shields inside the casing. Instead, the water velocity below the runner was 60% higher than the theoretical free fall velocity from the bucket exit [36]. Therefore, it was deduced that the interaction of the upper outflows from the Pelton runner with the casing walls had a great impact on the flow losses, while the interaction between the droplets and the casing below the runner was less significant. Droplets were also analyzed. The range of the particle diameter in the inner circle (the inner circle is defined as the circumference passing through the nozzles) ranged from 5 to 6 mm, while it was from 5.2 to 5.3 mm in zones with high velocity (10–11 m/s). This is in agreement with literature data, where a diameter of 5.26 mm was found as a common dimension for a stable water droplet at a free falling velocity of 10 m/s. Measurements in [45] showed that at 7.56 m³/s and 50 MW, the flow from the runner consisted mainly of water droplets with particle diameters from 3 to 12 mm, and the maximum velocity was detected directly beneath the runner in the order of 11.25 m/s. The maximum vertical component of droplet velocity during the impact below the lower buckets ranged from 10 to 11 m/s. Near to the casing walls the droplet velocity reduced to 4–6 m/s, that was also the range with the highest number of droplets (see Figure 6). However, the effect of droplet velocity on the aeration requirements was not investigated.

### 4.3 Counterpressure Pelton turbines

**Figure 7:**
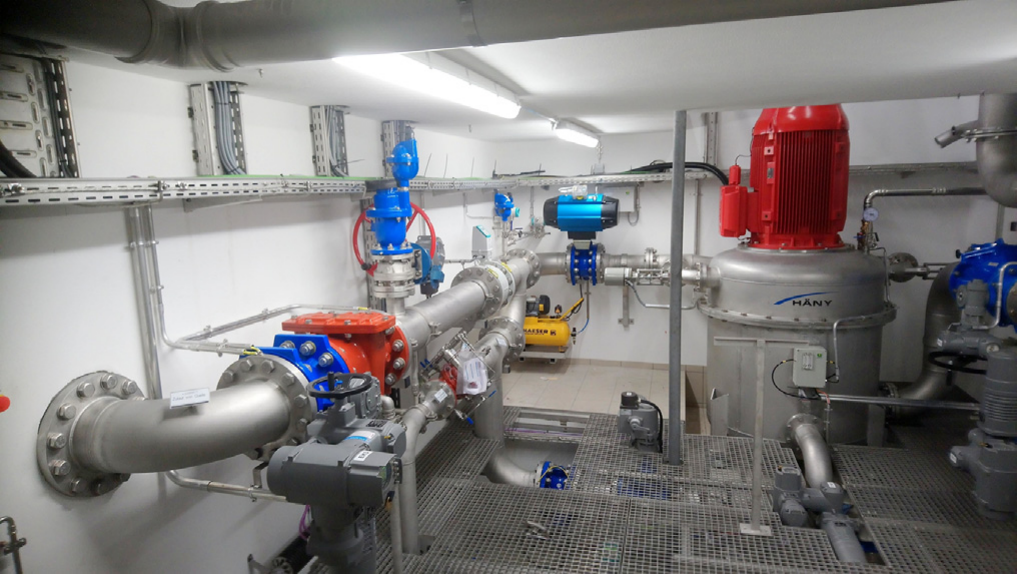
Counterpressure Pelton turbine, photo courtesy of Hirschi Lukas (Haeny company).

The complexity of the aeration increases for Pelton turbines working under counterpressure conditions. The counterpressure operation is generally adopted in cases of high water level oscillations at the tailrace, or when it is not possible to install the Pelton unit above the water basin, so that the Pelton unit is installed below the downstream water basin (underground). To carry the water to the above water basin, the casing is pressurized. In general, the counterpressure operation leads to a lower powerhouse cost because the powerhouse can be installed underground [2]. However, the cost of a counterpressure Pelton turbine may be higher than the cost of a Pump as Turbine (PAT), that is properly conceived to work with a counterpressure operation [46] (Figure 7).

During counterpressure operation, dissolved air entrainment and detrainment are expected to increase. Therefore, there is the challenge to better understand and predict the air that is detrained and transported to the tailrace, because this may cause corrosion and reduced transport capacity at the tailrace [41]. The detrainment of undissolved air depends on the operating condition and dimensions of the turbine (e.g. turbine power), while the entrainment and the detrainment of dissolved air depend both on the diffusion in the boundary layer at the interface water-air and on the fluid convection.

A correct understanding of the aeration process is thus relevant, especially for pumping hydropower plants and for applications in aqueducts, where Pelton units generally operate in counterpressure. In pumping hydropower plants, the air entrained below the Pelton runner has to be de-aerated in the tailrace to be used in pumping mode, to avoid pump efficiency losses and cavitation (for simultaneous operation of pumps and turbines, the water is conveyed directly from the tail water of the turbine to the pumps, see [25]). In water supply networks and reverse osmosis desalination plants the entrained air has to be maintained at a certain value [41]. In [41] a literature review is presented, including findings until 2017, with experimental tests to shed more light on the air detrainment process. It was found that the installation of air removal devices at high points of the system is recommended to reduce the surplus of dissolved entrained water, while design equations are presented to avoid detrainment of undissolved air.

In [26] the aeration process of the casing of a counterpressure Pelton turbine has been studied and optimized. The investigated turbine was a two-jet Pelton turbine with a vertical axis; the hydraulic head was *H* = 60 m and the design flow rate was *Q* = 4.3 l/s. Three different configurations were tested to test the air detrainment process: (a) no additional baffle plates and flow straighteners (inserts); (b) implementation of a baffle plate mounted on three deflectors; (c) implementation of flow straighteners with honeycomb-like structure. By option (b), the direct impingement of water jets and droplets from the Pelton buckets on the tailrace were prevented, while by option (c) turbulence was reduced. In configuration (b), the undissolved air demand was reduced by around 50%, while the solution (c) reduced the undissolved air demand from 93% to 99% of the original air demand.

An interesting case study is that reported in [2]. A Pelton turbine was installed underground (the site is situated in an area with landscape protection restrictions) and integrated with a compressor, able to cover the head difference between the tailrace water level of the Pelton turbine and the water level of the pressure interruption chamber. The gross head of the Pelton turbine is 181.95 m, and the net head is 160.40 m. The maximum turbine flow is 0.58 m^3^/s. Runner diameter is 697 mm, while casing diameter and height were 2200 mm and 2400 mm (including the counterpressure tank), respectively, in line with Eq. (1). The compressor power is 22.6 kW, and the height covered by the counterpressure is 5.5 m. The supplementary cost of the electromechanical equipment (hermetic casing, compressor system) was around 20% of the cost for a traditional Pelton turbine, but the global cost of the plant was lower because the powerhouse was not built.

Another case study worth mentioning is that developed at the Kopswerk II power plant in Austria, with a 180 MW Pelton turbine and a gross head ranging from 737 m to 804 m [47,48]. The pump power is 155 MW. Based on the lower reservoir water level, the Pelton turbine is below the water level of the lower reservoir by 1–16 m. When the vertical distance between the Pelton runner and the water level is 16 m, the casing was pressurized at 3 bar, and at 1 bar when the distance is 1 m. The characteristics of this counterpressure operation unit are:

• the Pelton turbine can be accessed through an upper cover, and the motor generator through the lower and upper cover.
• Two series of butterfly valves in order to provide air ventilation from the surge chamber.
• Lower and upper shaft-seals: the lower shaft seal is close to the generator, while the upper one is mounted above the pressure surge tank and below the radial turbine bearing.
• Two small and six main oil-free rotary screw compressors supply the compressed-air to the three units.

In [47, 48], in order to estimate the effects of the counterpressure (*CP*), a power loss analysis was performed to estimate ventilation and bearing losses. It was concluded that well positioned Pelton turbines with counterpressure can exhibit an increase in efficiency between 0.5% and 1% due to the gain of head.

The estimated ventilation losses were 240.9 kW (0.13% of the installed power) and 62.6 kW (0.03%) for *CP* = 3 bar and *CP* = 1 bar, respectively, that can be estimated by the equations proposed in [15] for vertical axis and horizontal axis turbines.

Bearing losses, attributed to the increase of axial forces due to bigger diameter of the two shaft seals and pressure in the tailrace water, were 160 kW (0.09%) and 42 kW (0.02%) at *CP* = 3 bar and *CP* = 1 bar, respectively. However, the gain of head was the dominant factor, so that the increase of the operating power ΔP could be estimated by Eq. (7).

(7) ΔP=1.88−0.157(CP−2)

**Figure 8:**
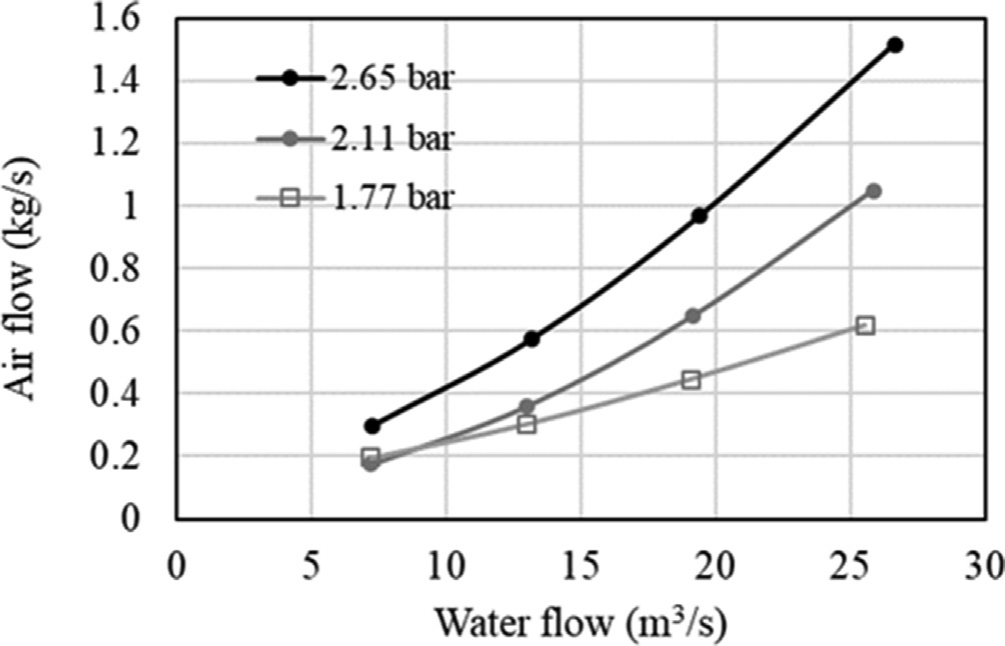
Air flow versus the water flow discharged by the Pelton turbine (elaborating data of [47]).

Instead, the required air can be calculated from Figure 8, and 90% of the air was estimated to be dissolved in the water. At water flows below 10 m^3^/s, the required air flow at the lowest pressure seems to overcome those required at the higher pressure, but this phenomenon was not investigated.

Until 2015, around 20 counterpressure Pelton units have been installed in water supply network, mainly at tank-inlets [41]. Some case studies can be found in the references, [49, 50, 51], for Switzerland and presented in Table 1. In Romano d'Ezzelino (Italy) a counterpressure Pelton turbine has been installed with a head difference of 50 m, 412,000 € of installation cost and 435,000 kWh/y of predicted generation^1^.

**Table 1:** Case studies of counterpressure Pelton turbines. ∗not specified if gross or net head.

	Available head (m) (net head)	Flow (l/s)	Power (kW)	Production (kWh/y)	Cost (CHF)
Gordola	252∗		33	200,000	450,000
Bellinzona	582 (569)	20	91	600,000	Not available
Morges	115 (105)	83	75	350,000	Not available
Bourg Saint-Pierre	466 (403)	34	111	739,000	Not available
Mels	415∗	13	41	Not available	Not available
Fällanden	140∗	16	17	Not available	Not available

## 5 Structural analysis: vibrations and weight

### 5.1 Complexity and research challenges

The structural design of the casing is of high importance to sustain vibrations, to support the loads and to resist the fluid-structure interaction related stresses. However, in literature, very limited studies have been found on the structural behavior of the casing, differently from the studies on the runner vibration, that can be found more frequently [52].

### 5.2 Literature review

Dhakan and Chalil (2013) [18] analyzed the structural design using Ansys Mechanical software. The operation frequency of the unit was 7.42 Hz and 4 main modal frequencies were found: bending mode at 105.85 Hz, torsion mode at 153.55 Hz, tensile stretching mode at 185.28 Hz and compressive mode at 222.60 Hz. The first mode of natural frequency occurred above 1.3 times of the runaway speed. However, it is not possible to generalize results, since turbine dimensions are not known. The casing design was optimized and a weight reduction of around 12% (weight reduction of 1770 kg) was achieved by testing different ribs and thicknesses, considering a minimum safety factor of 2 and an allowable deformation of 1.25 mm. The first frequency slightly increased to 110 Hz. It was found that the casing shell thickness had large impact on stress and deformation, and on the modal frequency: the first modal frequency halved when the casing thickness reduced from 45 mm to 10 mm. Above a certain value of shell thickness, increase of thickness did not affect so much stress and deformation. Addition of 12 vertical ribs and 2 horizontal ribs reduced stress and deformation, so that the shell thickness could be reduced from 35 mm to 25 mm with the weight reduction of 1770 kg. Considering a constant thickness of 25 mm, Von Mises maximum stresses reduced from 204 to 120 MPa by adding the ribs, the deflection reduced from 1.4 mm to 0.75 mm and the frequency increased from 65 Hz to 110 Hz.

In [53] vibration measurements were carried out in a double nozzle Pelton turbine, 4 MW power output, maximum discharge of 1.75 m^3^/s, rotational speed 500 rpm and 20 buckets. At the head of 303 m, the frequency peak was 285 Hz, that corresponds to 34 times the rotational turbine frequency (8.33 Hz) and 1.7 times the bucket passage frequency (166.67 Hz). The amplitude peak was 0.74 × 10^−4^ m.

### 5.3 Dataset collection and weight estimation

The weight of the casing depends on the casing dimensions and thickness. The width of the casing depends on the bucket dimensions, that depend on the jet diameter, thus the casing width is proportional to Qi2gH, where *Q* is the flow rate (m^3^/s), *i* is the number of jets and *H* (m) is the head. The other casing dimensions (those on the plane orthogonal to the axis) depend on the runner diameter. Therefore, it is possible to express the casing weight *G* (kN) as a function of the term *f* in Eq. (8):

(8) f=D2Qi2gH

**Figure 9:**
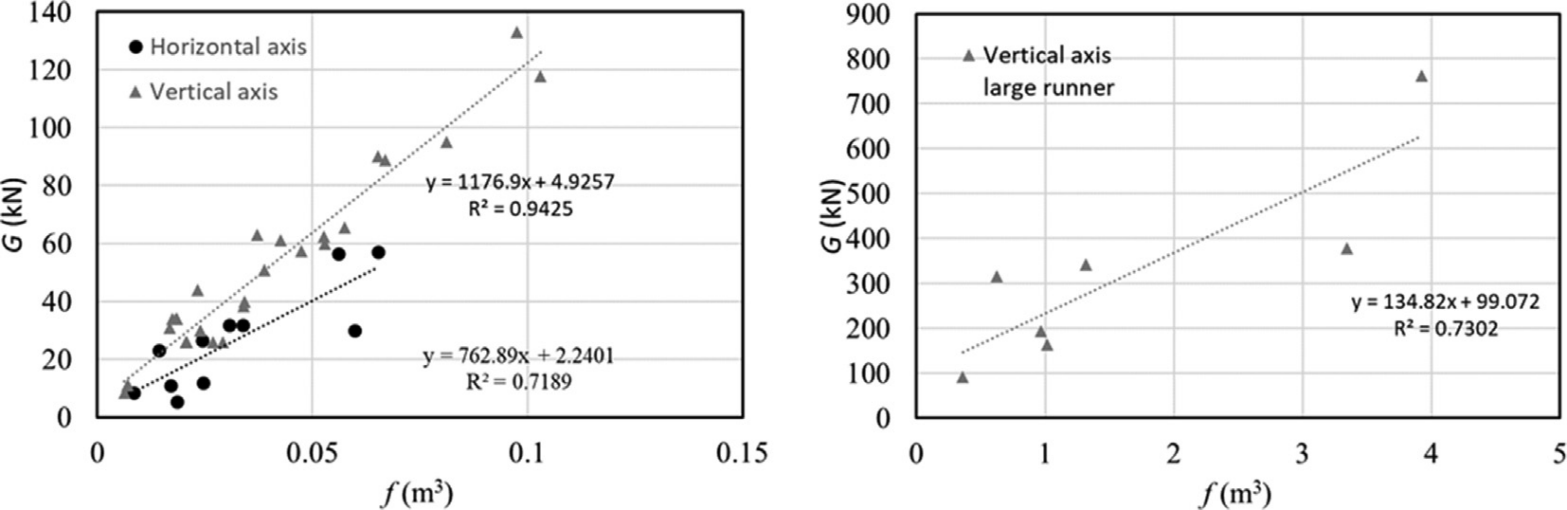
Weight of turbine casing versus *f* eq. (8)

In order to find the relation between the weight and the factor *f*, we collected data from three hydropower companies: Zeco Hydropower, Ghiggia Ingegneria di Impianti and Voith Hydro. Zeco Hydropower used the material S275JR (UNI EN 10025) for small Pelton turbines (7,800 daN/m^3^), Ghiggia Ingegneria S 235 JR for small Pelton turbines (7,850 daN/m^3^), and the big Pelton turbines manufactured by Voith Hydro were made of carbon steel (7,800 kg/m^3^). The data are listed in Table 2, and in Figure 9; the achieved equations can be used for a preliminary estimate of turbine casing weight *G* (kN). The casing of vertical axis units is heavier than the horizontal axis one, because it has to support the generator weight, and typically there are more jets, thus more structural connections. This analysis does not consider the thickness, that is assumed to be implicitly included in the already considered terms. In large hydro units, the design principle and the erection philosophy play a more dominant role than for small hydro, where the design philosophy is very similar in each plant.

**Table 2:** List of casings from Ghiggia Ingegneria di Impianti (G.I.I.), Zeco Hydropower (Zeco) and Voith Hydro. In the column of ‘Axis’, H indicates the horizonal axis and V indicates the vertical axis.

Head *H* m	Flow *Q* m^3^/s	Diameter *D* m	Jets *i*	Weight *G* kN	Axis	Company
125	0.15	0.58	1	5.5	H	G.I.I.
145	0.36	0.65	2	12	H	G.I.I.
322	0.7	0.95	2	30	H	G.I.I.
387	0.4	0.800	2	32	H	Zeco
520	0.85	0.930	2	56.5	H	Zeco
164.6	0.28	0.700	2	26.5	H	Zeco
405	0.5	0.800	2	32	H	Zeco
130	0.2	0.620	2	11	H	Zeco
98	0.15	0.450	2	8.5	H	Zeco
507.7	0.275	0.620	2	23	H	Zeco
398.4	1.1	0.910	2	57	H	Zeco
120	1.2	0.73	6	40	V	G.I.I.
120	2.2	1.040	5	118	V	Zeco
115	0.55	0.585	4	34	V	Zeco
262	0.7	0.645	4	26	V	Zeco
155	0.75	0.680	4	26	V	Zeco
165	0.9	0.680	4	26	V	Zeco
120.4	1.25	0.895	5	65.5	V	Zeco
157.8	1.25	0.840	4	60	V	Zeco
183	1.5	0.920	4	89	V	Zeco
174.12	1.9	0.900	5	90	V	Zeco
146.23	2.25	0.985	6	95	V	Zeco
191.65	1.3	0.755	5	63	V	Zeco
121	0.55	0.585	4	34	V	Zeco
115	2.5	1.020	6	133	V	Zeco
122.29	0.308	0.440	5	11	V	Zeco
120	0.51	0.585	4	34	V	Zeco
222.5	0.95	0.805	4	51	V	Zeco
250	0.7	0.645	4	26	V	Zeco
116.5	1	0.725	5	38.5	V	Zeco
88.6	0.7	0.635	5	44	V	Zeco
142	0.7	0.645	4	30	V	Zeco
252.8	0.18	0.420	2	8.5	V	Zeco
430	1	0.840	3	61	V	Zeco
198.3	0.6	0.585	4	31	V	Zeco
138	1.14	0.800	4	57.5	V	Zeco
263	2	0.88	6	62.5	V	Zeco
171	0.4	0.44	5	11	V	Zeco
1150	13.5	2.878	6	164.3	V	Voith
487	32.5	3.77	6	378.45	V	Voith
700	12.5	2.69	6	195.04	V	Voith
615	32	4.22	6	763.5	V	Voith
1220	10.6	2.42	6	315.45	V	Voith
746	6.2	1.885	5	92	V	Voith

## 6 Discussion, open questions and challenges

### 6.1 Open questions

Casing design plays an essential role to achieve a good overall efficiency of Pelton units. However, the internal hydrodynamics is very complex and affects all the related behavior that should be taken into account during operation: fluid structure interaction, aeration, vibrations and deformation. However, very limited attention has been given to the casing design and optimization.

To investigate the hydrodynamics inside the casing, experimental tests were not found suitable and effective because the air in the casing becomes rapidly saturated, hiding flows and jets far from the casing walls [36]. In Larsen (2015) [54] different ways to detect flow have been tested; however, the obtained images did not enable quantitative analyses of the flow. Therefore, CFD simulations should be used to analyze the flow inside the casing, but very few studies can be found in the literature, due to the high computational costs related to the multiphase and unsteady flow, 3D phenomena and high turbulent behavior. The flow in Pelton turbines has therefore not been analyzed so far with such detail as the flow in the reaction turbines, and the understanding of the physics of key phenomena is weak [55]. Therefore, casing flow behavior is still challenging to be understood. In the near future, Eulerian mesh based CFD solvers are going to be used more easily to investigate the flow behavior [27], despite the difficulty of reproducing the flow coming out from the buckets. Lagrangian techniques conceptually represent an alternative strategy [56]. Full scale simulations might become more affordable, and injectors, complete runner, turbine casing and multi jet operation could be easily simulated, while nowadays a lot of CFD studies do not include the casing in the simulations [57]. The deeper understanding of flow behavior allows to optimize the design and to quantify with CFD analysis the impact of the design modifications on the performances. Moreover, as the need of modernization increases [58, 8], CFD analysis allow to predict damages of existing hydraulic Pelton turbine components, as distributors and casings are often concrete-embedded in large units [56].

The aeration in hydropower plants affects oxygen content downstream. A non-optimal air content can affect water quality in drinking water systems and fish life [59]. The aeration also affects the performance of the turbine. In Pelton turbines, ventilation losses, that depend on fluid density inside the turbine casing (a mixture of air and water), affect both the power losses during runner rotation and the quality of the jet [60]. The aeration process is of extreme importance especially for Pelton turbines operating with a counterpressure. However, aeration has been only a little investigated, and few studies can be found in literature. More studies should be performed to investigate how the aeration is affected by the casing geometry and design, which depend on the fluid dynamic interaction.

Vibration and deformation also represent important topics, strongly dependent on the internal hydrodynamics, but information are not exhaustive, as for example those available for Francis turbines [61, 62]. The few studies that were here discussed did not allow to generalize results, but somehow, we tried to generalize them to give engineering suggestions So far, Pelton turbines are designed such that the natural frequency is beyond the frequency of runaway speed, and the turbines are safe. However, continuous reduction of material [63] and size may result in overlapping of natural frequency with the runner frequency and may lead to resonance. When the components are assembled, the global natural frequency drops. This frequency is not known and cannot be determined in early design phase. It can only be determined after the complete installation of the turbine: this is big challenge for the designers. The reduction of the casing weight is also important to reduce cost, and the use of ribs and shroud can allow to reduce thickness, i.e. the total weight. In light of this, equations are proposed to estimate the weight of casing, and, based on previous study on weight reduction, it is reasonable to think that the estimated weight can be reduced by 12% by ad hoc structural analysis.

Control algorithms for Pelton turbines are also important research topics under investigation [64, 65].

### 6.2 Comparison with other turbines

The CFD simulation of the casing of Banki and Turgo turbines is also challenging. In [66] the number of grid elements of the Banki casing for the CFD simulations were 7 times more than in the other domains (e.g. the runner). Instead, Turgo turbines exhibit the advantage of outflow from the opposite direction than the injectors, but, nevertheless, water which is rebounded from the casing walls can interfere with the jets and the runner, worsening the performance [11] and increasing ventilation losses [67].

The aeration system is not of interest only for Pelton turbines. The auto-venting turbines (AVT) have been introduced to optimize air distribution at the tailrace, reducing impacts on the ecosystems downstream [59]. AVTs can be used both in new projects and in retrofitting projects, increasing the power plant efficiency, as highlighted in [68]. In one case it was found an efficiency increase by 2% without aeration, and 1% with an AVT with peripheral aeration, and in a second case an efficiency improvement by 3.8% without aeration, and 1% with an AVT with distributed aeration. Similar results were obtained with central aeration, increasing the power output by 21%/11% without/with aeration. March and Fisher (1999) [69] reported that the weighted efficiency of a Francis turbine increased by 3.7% after the replacement of the turbine runner with a new AVT, increasing the turbine capacity by 10%. Three more cases are reported in [68].

Weight-related discussions on water wheels and Francis and Kaplan turbines are provided in [70].

### 6.3 Design guidelines

Equations to preliminary design the casing of Pelton turbines are summarized in Eqs. (1), (2), (3), (4), (5), and (6), while the weight can be estimated by the equation presented in this work and reported in Figure 9. Dimensions can be optimized, and the thickness reduced by including in the casing inserts and shrouds, with the aim of making the casing lighter and to reduce the splashes and water jet interferences. The key studies that recommend how to design the internal shrouds and ribs are here discussed, but CFD simulations are recommended to further predict the hydraulic behavior of the casing, in order to overcome limitations of experimental measurements [71], especially for multi-nozzle turbines [72]. The additional complexity is that the casing is a component which may not be symmetric in general, and this requires the simulation of the full domain when the flow behavior inside the casing is of interest.

Only two studies were found on casing vibration and deformation, thus the generalization should be taken with care due to the few available data. This should stimulate future research, while studies on the runner vibration can be found more easily in literature [73, 74].

## 7 Conclusions

The casing of a Pelton turbine affects the performance of the power plant, particularly efficiency, aeration and structural stresses. It was found that an optimal width can increase the efficiency by 3% (this result cannot be generalized, but it shows as it is possible to improve the unit efficiency by acting on the casing), while baffles and side shrouds can improve the performance by 0.5% and 2%, respectively. However, the losses depend on the specific speed and on the type of the Pelton turbine (axis orientation). Therefore, there is the need of more exhaustive guidelines that guide the designer in choosing the optimal location and geometry of baffles and inserts, to improve the internal hydrodynamics, and the results discussed in this review provide preliminary guidelines.

Both Smooth Particle Hydrodynamic method [37] and Eulerian CFD methods (Petley et al. works) have been carried out, but more numerical and computational efforts should be done to reduce computational costs of these methods, since they involve complex 3D, unsteady and turbulent phenomena, with air inclusion, free surface thin jets and droplets.

Aeration is an important matter to consider for reducing downstream impacts on water quality and reducing ventilation losses, although ventilation losses are generally limited <0.1% of the produced power.

Studies on structural behavior should be devoted to better provide guidelines for vibration estimation, like the dominant casing frequency. Few scientific studies have been found in literature, while large turbine manufactures perform on a regular basis structural studies and field measurements. One of the major challenges is the detuning the runners in the field. In one study, the dominant frequency was found to be 34 times the rotational turbine frequency (8.33 Hz) and 1.7 times the bucket passage frequency, but with only one study it is not possible to consider this a generalizable result. The global mode frequency can only be determined after the complete installation of the turbine: this is big challenge for the designers. Structural analyses are also useful to optimize the casing shape and reduce the weight. The empirical equations here proposed to estimate the weight of the casing add a piece of knowledge in this context.

Three clear goals for casing design are: (1) fluid dynamic optimization, (2) improve aeration and recover oxygen content, while reducing detrained air in counterpressure operation, and (3) structural integrity, with more specific related guidelines. It is also projected that Pelton turbines can be used to mitigate the flexible energy demand. This means that the turbine will undergo frequency load variation and start-stops. This will impact the structural integrity of the entire electro-mechanical structure, including the casing. Hence, comprehensive approach of turbine design is essential, especially in light of the crucial role of hydropower in the future [75].

## Declarations

### Author contribution statement

All authors listed have significantly contributed to the development and the writing of this article.

### Funding statement

This research did not receive any specific grant from funding agencies in the public, commercial, or not-for-profit sectors.

### Data availability statement

Data included in article/supp. material/referenced in article.

### Declaration of interests statement

The authors declare no conflict of interest.

### Additional information

No additional information is available for this paper.

## Footnotes

1 https://www.etraspa.it/area-stampa/comunicati-stampa/funzione-romano-dezzelino-una-centrale-idroelettrica-unica-italia-nel.
